# 3-Phenylpropan-1-Amine Enhanced Susceptibility of Serratia marcescens to Ofloxacin by Occluding Quorum Sensing

**DOI:** 10.1128/spectrum.01829-22

**Published:** 2022-08-16

**Authors:** Lujun Yin, Ping-Ping Zhang, Wei Wang, Shi Tang, Shi-Ming Deng, Ai-Qun Jia

**Affiliations:** a Key Laboratory of Tropical Biological Resources of Ministry of Education, School of Pharmaceutical Sciences, Hainan University, Haikou, China; b One Health Institute, Hainan University, Haikou, China; Johns Hopkins University School of Medicine

**Keywords:** 3-phenylpropan-1-amine, *Serratia marcescens*, quorum sensing, virulence factors, biofilms, ofloxacin

## Abstract

Serratia marcescens (S. marcescens) is an environmental bacterium that causes infections with high morbidity and mortality. Notably, infections caused by multidrug-resistant S. marcescens have become a global public health issue. Therefore, the discovery of promising compounds to reduce the virulence of pathogens and restore antibiotic activity against multidrug-resistant bacteria is critical. Quorum sensing (QS) regulates virulence factors and biofilm formation of microorganisms to increase their pathogenicity and is, therefore, an important factor in the formation of multidrug resistance. In this study, we found that 3-phenylpropan-1-amine (3-PPA) inhibited S. marcescens NJ01 biofilm formation and virulence factors, including prodigiosin, protease, lipase, hemolysin, and swimming. The combination of 3-PPA (50.0 μg/mL) and ofloxacin (0.2 μg/mL) enhanced S. marcescens NJ01 sensitivity to ofloxacin. Based on crystalline violet staining, scanning electron microscopy (SEM), and confocal laser scanning microscopy (CLSM), 3-PPA (50.0 μg/mL) reduced S. marcescens NJ01 biofilm formation by 48%. Quantitative real-time PCR (qRT-PCR) showed that 3-PPA regulated the expression of virulence- and biofilm-related genes *fimA*, *fimC*, *bsmB*, *pigP*, *flhC*, *flhD*, and *sodB*. Liquid chromatography-tandem mass spectrometry (LC-MS/MS) indicated that 3-PPA affected intracellular metabolites of S. marcescens NJ01, leading to reduce metabolic activity. These results suggested that 3-PPA inhibits the pathogenicity of S. marcescens NJ01 by occluding QS. Thus, 3-PPA is feasible as an ofloxacin adjuvant to overcome multidrug-resistant S. marcescens and improve the treatment of intractable infections.

**IMPORTANCE** Multidrug-resistant bacteria have become a major threat to global public health, leading to increased morbidity, mortality, and health care costs. Bacterial virulence factors and biofilms, which are regulated by quorum sensing (QS), are the primary causes of multidrug resistance. In this study, 3-PPA reduced virulence factors and eliminated biofilm formation by inhibiting QS in S. marcescens NJ01 bacteria, without affecting bacterial growth, thus restoring sensitivity to ofloxacin. Thus, the discovery of compounds that can restore antibiotic activity against bacteria is a promising strategy to mitigate multidrug resistance in pathogens.

## INTRODUCTION

Serratia marcescens (S. marcescens) is a Gram-negative bacillus belonging to the family *Enterobacteriaceae*, with its pathogenicity in humans first noted in 1913 (1). It is widely present in the air, soil, water, plants, and medical devices, and is an important opportunistic mutagenic pathogen that poses a threat to human and animal health (2, 3). It is implicated in septicemia, conjunctivitis, pneumonia, urinary tract infections, and meningitis (4), and can cause significant outbreaks of nosocomial infections. Furthermore, S. marcescens is a recognized cause of hospital-acquired infections, especially in immunocompromised patients, with increased infections noted in elderly and neonatal patients and with the use of certain invasive devices (1, 5).

Antibiotics have dramatically reduced deaths caused by S. marcescens infections. However, their overuse and misuse have also resulted in the emergence of antibiotic resistance (6). At present, S. marcescens has shown resistance to most antibiotics, except imipenem and tetracyclines (CLSI, 2020). The infectivity of S. marcescens and its inherent resistance to antibiotics, especially via the production of plasmids and efflux pumps, have greatly contributed to the emergence of multiple drug-resistant strains, ultimately complicating the treatment process (7, 8). The rate of antibiotic resistance is increasing, and the underlying mechanism is complex. Thus, the emergence of multidrug-resistant S. marcescens strains has become a critical issue for infection control.

At present, the World Health Organization (WHO) has identified bacterial resistance to antibiotics as the third most significant threat to human health, and there is an urgent need to develop alternative treatment strategies that can effectively eliminate antibiotic misuse. While free-floating S. marcescens bacteria are more susceptible to antibiotics, they can reorganize into structurally complex clusters composed of a self-synthesizing extracellular polymer matrix (biofilm), which is resistant to many antimicrobial agents (9, 10). Quorum sensing (QS) is a bacterial density-dependent gene expression system involving the binding of receptors and autoinducers to control the swarming, swimming, and biofilm formation of pathogenic bacteria through the production of virulence factors, which can lead to a decrease in antibiotic effectiveness (10, 11).

Serratia marcescens can secrete virulence factors, including DNase, prodigiosin, lipase, hemolysin, proteases, chitinase, and biosurfactants, and form biofilms through QS regulation (12, 13). QS-related gene mutants of S. marcescens can reduce virulence factor production, such as prodigiosin level and biofilm biomass, thereby decreasing pathogenicity (14). Therefore, interference in QS systems is a compelling alternative for attenuating pathogenicity and protecting the host against infection. In the current study, we used 3-phenylpropan-1-amine (3-PPA) as an antibiotic adjuvant to interfere with QS and interrupted signaling among bacteria without posing a threat to the microorganism, thus potentially solving pathogenic bacterial resistance.

We previously showed that hordenine possessed virulence suppression activity against S. marcescens by downregulating the expression of QS-related genes (15). Because prodigiosin production is regulated by QS, QS inhibitor (QSI) drugs were screened and obtained based on the inhibition of prodigiosin production. In this study, hordenine analogs were screened by observing their inhibitory effects on the prodigiosin production of S. marcescens. Therefore, we found that 3-PPA exhibited QS inhibitory activity against S. marcescens NJ01. This work provided a strategy to circumvent multidrug-resistant S. marcescens and to improve treatment outcomes of recalcitrant infections.

## RESULTS

### MICs and growth profiles.

**FIG 1:**
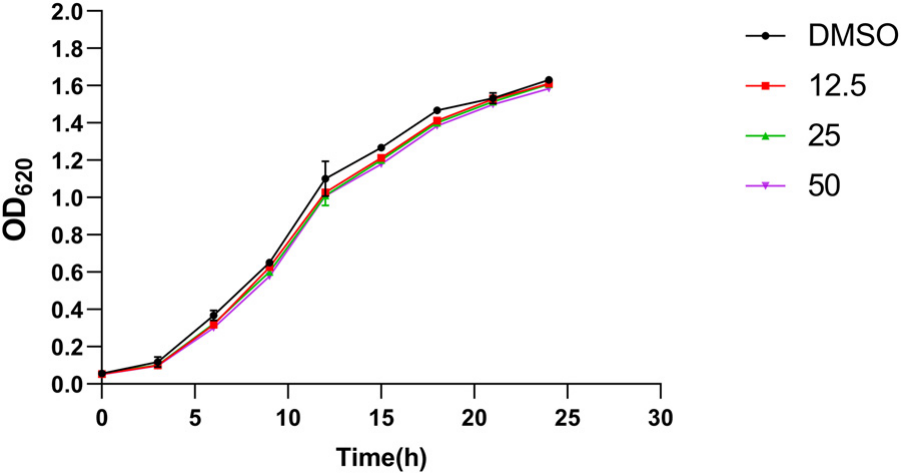
Growth curve of S. marcescens NJ01 treated with 3-PPA.

The MICs of 3-PPA and ofloxacin against S. marcescens NJ01 were 1,300 μg/mL and 4 μg/mL, respectively. The current study aimed to ensure that 3-PPA did not affect bacterial growth, but rather interfere with key cellular processes, and thus restore the sensitivity of bacteria to antibiotics. Therefore, we chose 3-PPA concentrations (12.5, 25.0, and 50.0 μg/mL) below the MIC (1 300 μg/mL) for testing. The growth profile of S. marcescens NJ01 showed that 3-PPA did not affect bacterial growth in the concentration range of 12.5 to 50.0 μg/mL (Fig. 1), in accordance with the aim of not directly killing the pathogen. Our results also showed that the MIC of ofloxacin was 4 μg/mL and ofloxacin had no bactericidal effect on S. marcescens NJ01 at 0.2 μg/mL (below the MIC). Therefore, we chose this concentration (0.2 μg/mL) in combination with 3-PPA (50.0 μg/mL) to show that 3-PPA could be used as an antibiotic adjuvant to restore sensitivity.

### 3-PPA suppressed prodigiosin, protease, lipase, and hemolysin secretion and inhibited motility and swimming ability.

**FIG 2:**
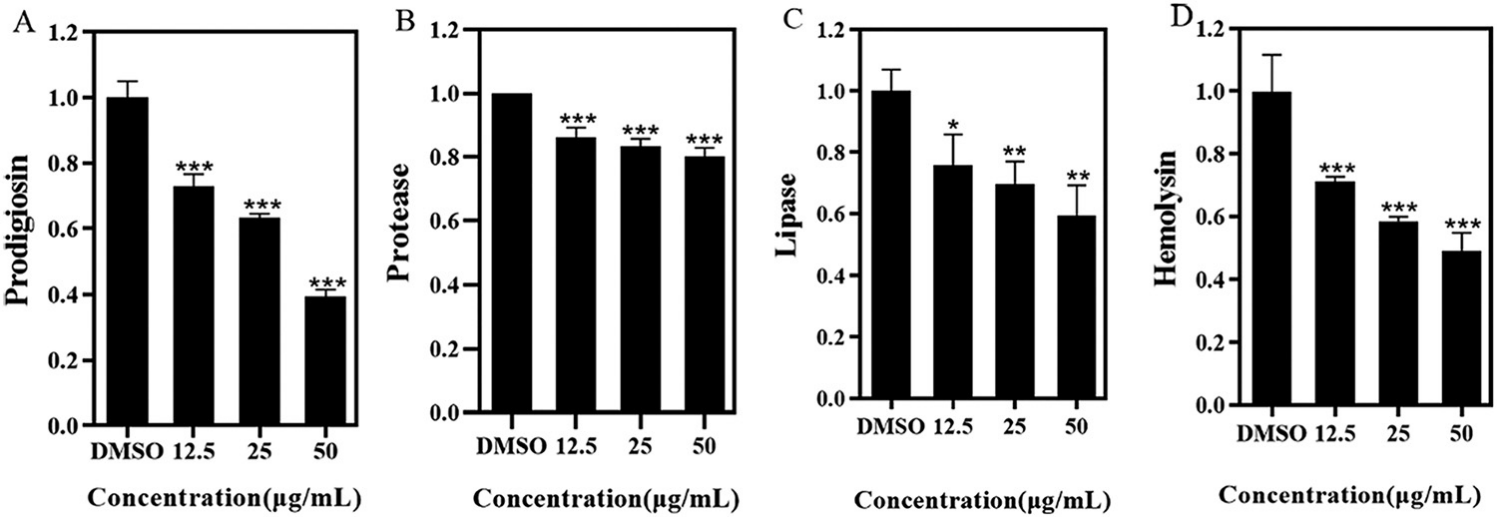
Inhibitory effects of 3-PPA on virulence factor production. (A) Inhibition of prodigiosin treated with 3-PPA; (B) Inhibition of protease treated with 3-PPA; (C) Inhibition of lipase treated with 3-PPA; (D) Hemolysin levels treated with 3-PPA.

**FIG 3:**
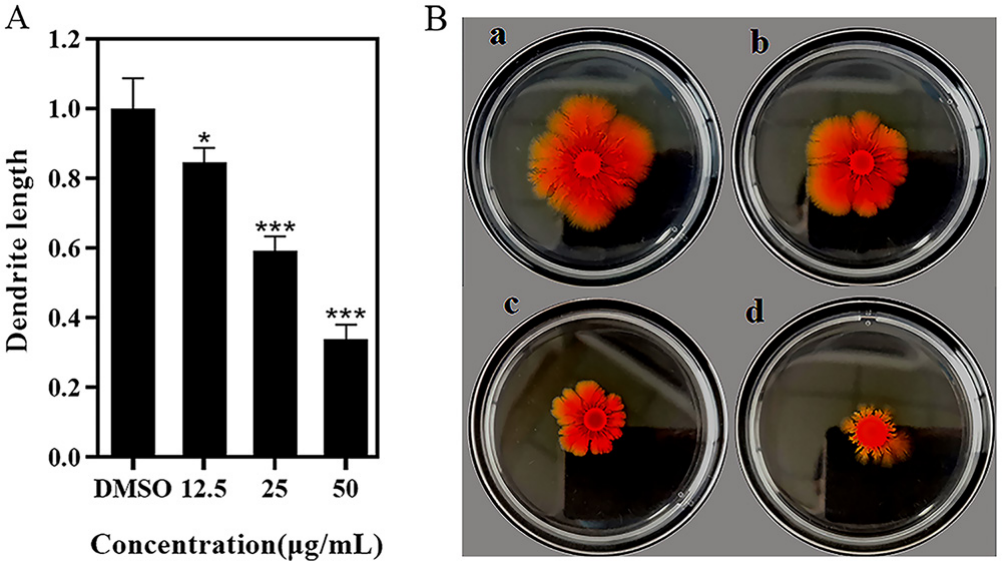
Effects of 3-PPA on swimming motility of S. marcescens NJ01. (A) Swimming motility of S. marcescens NJ01 under 3-PPA; (B) DMSO(a); 12.5 μg/mL 3-PPA treatment (b); 25.0 μg/mL 3-PPA treatment (c); and 50.0 μg/mL 3-PPA treatment (d).

Prodigiosin, a red pigment produced by S. marcescens NJ01, is essential for invasion, survival, and pathogenicity (16). As seen in Fig. 2A, prodigiosin production showed a content-dependent decrease with increasing 3-PPA, with a decline of 60% under 50.0 μg/mL of 3-PPA. Protease, an important virulence factor controlled by QS, can enhance the innate immune response of the host and inhibit protease secretions (17). As shown in Fig. 2B, 3-PPA (50.0 μg/mL) treatment resulted in a 20% inhibition of protease compared with the DMSO group. Lipase is involved in degrading phospholipid bilayers and mediating cellular signaling pathways in the host (18). As shown in Fig. 2C, lipase levels were reduced by 40% under 50.0 μg/mL 3-PPA. Hemolysin is another virulence factor secreted by S. marcescens NJ01. As shown in Fig. 2D, hemolysin production declined by more than 50% under 50.0 μg/mL 3-PPA treatment. In addition, the effect of 3-PPA on S. marcescens NJ01 motility was evaluated. As seen in Fig. 3B, the swimming ability of S. marcescens NJ01 was inhibited by 3-PPA, with a decrease of 65% under 50.0 μg/mL treatment (Fig. 3A).

### 3-PPA suppressed biofilm formation.

**FIG 4:**
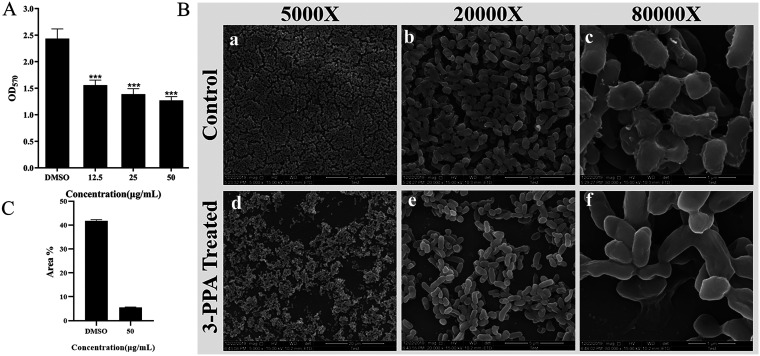
Inhibitory effects of 3-PPA on biofilm formation. (A) Quantitative analysis of biofilms treated with different concentrations of 3-PPA; (B) Inhibition of biofilm formation by 3-PPA (50.0 μg/mL) by SEM analysis; DMSO group (magnify 5,000×; (a)); DMSO group (magnification, 20,000×; (b)); DMSO group (magnification, 80,000×; (c)); 3-PPA treated group (magnification, 5,000×; (d)); 3-PPA treated group (magnification, 20,000×; (e)); 3-PPA treated group (magnify 80,000×; (f)); (C) Quantitative analysis of biofilm formation in 5,000× group.

Bacteria form highly complex structures, i.e., biofilms, which can withstand adverse external stimuli and are an important cause of drug resistance. The inhibitory effects of 3-PPA on biofilm formation were analyzed by crystalline violet staining. As shown in Fig. 4A, compared with the DMSO group, 3-PPA inhibited biofilm formation and the inhibitory effect increased with increasing drug concentration. Notably, at 50.0 μg/mL, 3-PPA treatment reduced biofilm formation by 48%.

**FIG 5:**
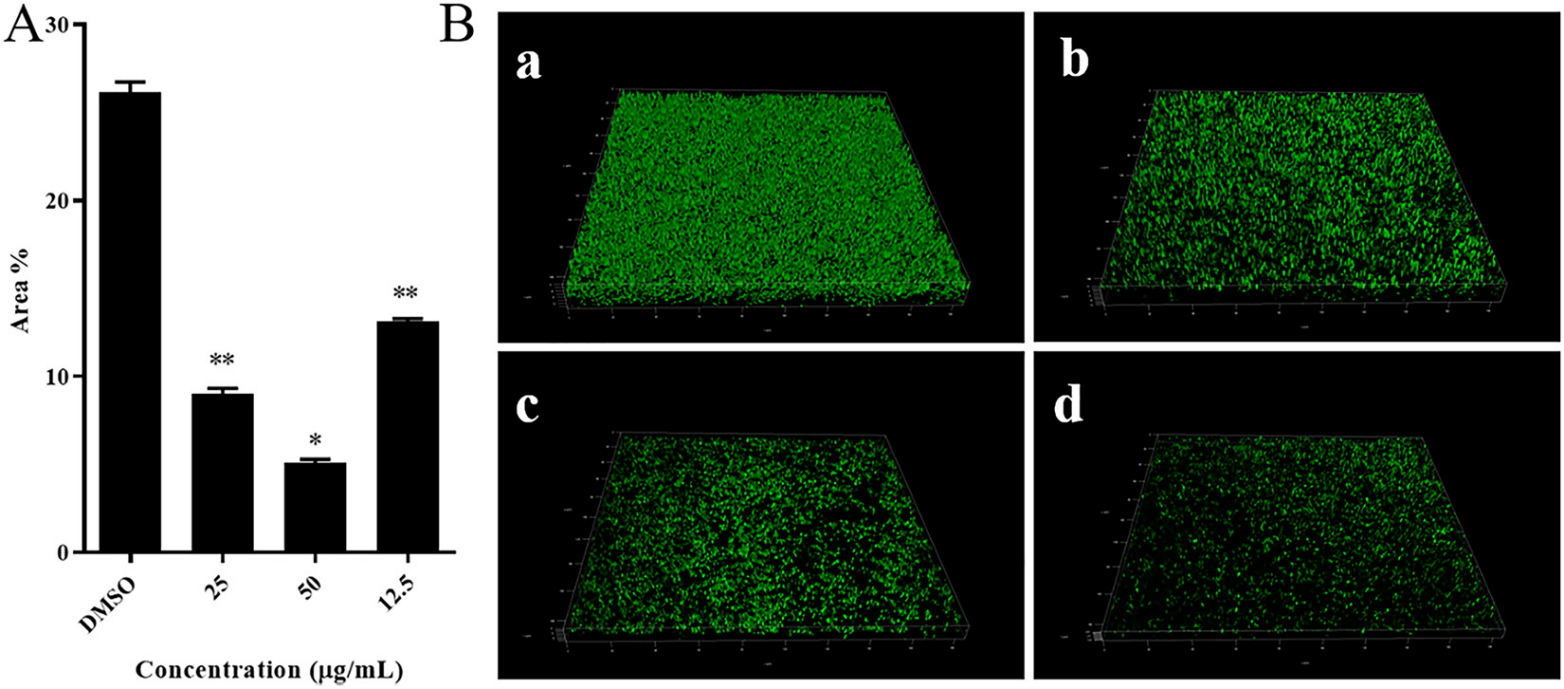
CLSM characterization of inhibitory effects of different concentrations of 3-PPA on S. marcescens NJ01 biofilm formation. (A) Area of S. marcescens NJ01 biofilm formation under treatment with different concentrations of 3-PPA; (B) DMSO (a); 12.5 μg/mL 3-PPA treatment (b); 25.0 μg/mL 3-PPA treatment (c); and 50.0 μg/mL 3-PPA treatment (d).

Visual confirmation of the three-dimensional (3D) structures of the biofilm treated with 3-PPA was observed using SEM. As shown in Fig. 4B, the blank DMSO group biofilm was a dense net-structured system connected by fibrous structures. However, the S. marcescens NJ01 biofilm was impacted by 3-PPA treatment at 50.0 μg/mL with a reduced fiber structure, scattered appearance, and compromised integrity. We further assessed the effect of 3-PPA on biofilm formation using CLSM. As seen in Fig. 5B (a), the blank DMSO group biofilm accounted for 26% of the area as quantified by ImageJ (v1.8.0.112) (Fig. 5A). In contrast, biofilm area decreased to 13%, 9%, and 5% after exposure to 3-PPA at 12.5, 25.0, and 50.0 μg/mL, respectively Fig. 5B (b–d). Thus, the S. marcescens NJ01 biofilm structure became looser and thinner following exposure to high concentrations of 3-PPA (Fig. 5A).

### 3-PPA combined with ofloxacin erased formed biofilms.

**FIG 6:**
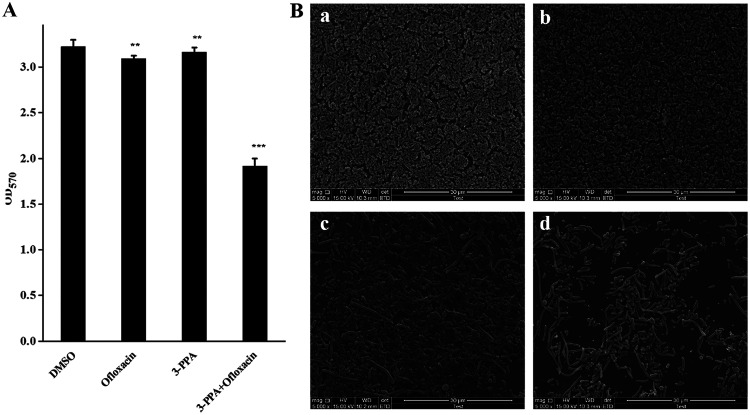
Disruption of preformed biofilms of 3-PPA combined with ofloxacin. (A) Quantitative analysis of formed biofilm. (B) SEM characterization of damage to preformed biofilm; DMSO (a); 50.0 μg/mL 3-PPA treatment (b); 0.2 μg/mL ofloxacin treatment (c); and 50.0 μg/mL 3-PPA combined with 0.2 μg/mL ofloxacin treatment (d).

Given the inhibitory effects of 3-PPA on biofilm formation, we also explored whether 3-PPA can increase the sensitivity of biofilms to traditional antibiotics. Thus, biofilms were exposed to 3-PPA and antibiotics in combination. 3-PPA (50.0 μg/mL) and ofloxacin (0.2 μg/mL) alone had weak effects on biofilm erasure, but relatively strong effects when in combination, with a biofilm erasure rate of 44% (Fig. 6A). The biofilm 3D structure was observed by SEM analysis (Fig. 6B). As seen in Fig. 6B (a), the blank DMSO group biofilm was a tightly connected layer with a network structure. Fig. 6B (b and c) show the biofilms after treatment with 3-PPA and ofloxacin, respectively. Combined treatment with 3-PPA and ofloxacin resulted in the significant dispersal, destruction, and reduction of the preformed biofilm Fig. 6B (d).

### 3-PPA reduced S. marcescens NJ01 pathogenicity by down-regulating QS-related gene transcription.

**FIG 7:**
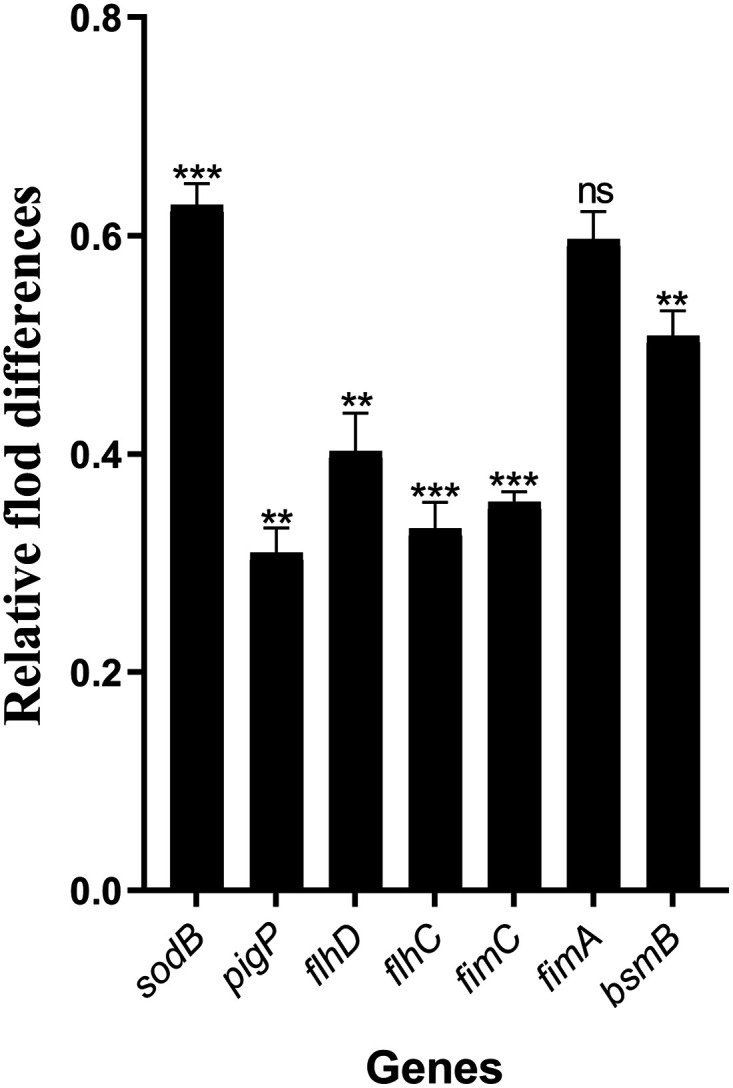
3-PPA treatment downregulated expression of QS-related genes in S. marcescens NJ01.

The quantitative real-time PCR (qRT-PCR) analysis was performed to assess the mechanisms underlying the inhibitory effects of 3-PPA on biofilms and virulence factors. We analyzed the effects of 3-PPA on the transcript levels of *fimA*, *fimC*, *pigP*, *flC*, *flhD*, *bmsB*, and *sodB* genes, which are involved in S. marcescens NJ01 hyphal production, adhesion, motility, and biofilm formation (19). As shown in Fig. 7, the expression levels of *fimA* and *fimC*, which are related to biofilm formation (20), were inhibited by 40% and 64%, respectively, following exposure to 50.0 μg/mL 3-PPA. The expression of *pigP*, which is associated with the synthesis of prodigiosin (21), was downregulated by 69%. The *flhC* and *flhD* genes, which are related to bacterial motility (22), were downregulated by 67% and 60%, respectively. As an essential virulence gene, *bsmB* is controlled by QS and participates in biofilm assembly, lipase, protease secretion, and S-layer protein production (10). As shown in Fig. 7, 3-PPA downregulated the expression of *bsmB* by 50%. These results agree with those above, suggesting that 3-PPA acts on virulence factors. We also investigated the expression of genes related to detoxification enzymes and found a 37% inhibition in the expression of *sodB*, which encodes superoxide dismutase (SOD) (Fig. 7).

### 3-PPA treatment induced disorder in intracellular metabolites of S. marcescens NJ01.

**FIG 8:**
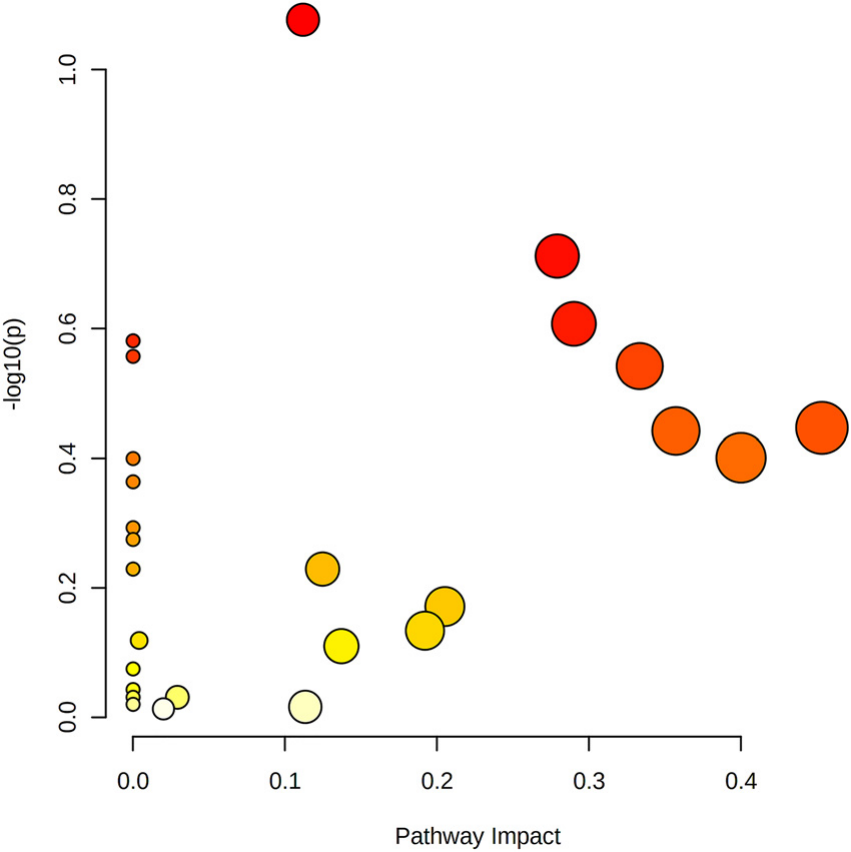
Metabolic pathway analysis of S. marcescens NJ01under 3-PPA treatments.

We used LC-MS/MS to investigate changes in intracellular metabolites in S. marcescens NJ01 after 3-PPA exposure (Fig. S2 in Supplemental File 1). The metabolic differentiators were screened by SIMCA v14.1 (Umetrics Company, Sweden). Principal component analysis (PCA) showed differences between the 3-PPA and control groups (Fig. S2A in Supplemental File 1). An orthogonal projection to latent structure discriminant analysis (OPLS-DA) model was developed, and the R^2^ and Q^2^ values in permutation analysis of OPLS-DA were less than 1 (Fig. S2B in Supplemental File 1), proving that the OPLS-DA model was robust and did not overfit. The S-plot and variable importance projection (VIP) values in the OPLS-DA (Fig. S2C in Supplemental File 1) and volcano plots (Fig. S2D in Supplemental File 1) were screened for metabolic differentiators. In total, 130 metabolic differentiators (Table S1 in Supplemental File 1) were finally screened by combining ploidy changes and magnitudes of significance. The hierarchical cluster analysis heatmap (Fig. S3 in Supplemental File 1) visualized the high and low expression levels of metabolic differentiators in each sample by color. Fig. S3 in Supplemental File 1 shows that metabolites that were high in the control decreased after 3-PPA treatment. In total, 14 metabolic differentiator pathways were retrieved by KEGG pathway analysis (http://www.kegg.jp/kegg/pathway.html), including alanine, aspartate, glutamate metabolism, butanoate metabolism, sphingolipid metabolism, and arginine biosynthesis, etc. (Fig. 8 and Table S3 in Supplemental File 1).

## DISCUSSION

When the density of a microbial population in the environment reaches a certain threshold, it can affect the expression of specific genes and modulate microbial physiological characteristics, such as bioluminescence, antibiotic synthesis, and biofilm formation (23–25). Using this QS phenomenon of “cell-to-cell communication,” microorganisms can coordinate within complex environments, produce virulence factors, and promote biofilm formation as part of the pathogenic process (26), which may also lead to drug resistance.

Serratia marcescens NJ01 can produce multiple QS-related virulence factors, including prodigiosin, hemolysin, protease, lipase, swarming motility, and biofilms. Biofilms and virulence factors can further increase the resistance of bacteria to antimicrobial agents.

Interfering with the bacterial network without affecting the viability of target bacteria is considered an important alternative strategy to combat bacterial infection while reducing the risk of selection pressure (27). This strategy may provide potent alternative pathways of anti-infective action, thereby attenuating bacterial resistance to antibiotics (28, 29). In this study, 3-PPA showed QS inhibitory activity against the tomato pathogen S. marcescens NJ01. The MIC of 3-PPA was 1,300 μg/mL, thus we selected three concentrations (12.5, 25.0, and 50.0 μg/mL) below the MIC for further study of its inhibitory effects on biofilms and virulence factors regulated by QS. S. marcescens NJ01 produced multiple QS-related virulence factors, including prodigiosin, hemolysin, protease, lipase, and swarming motility. Interference with virulence factors may be an efficient approach for attenuating S. marcescens NJ01 pathogenicity. Our results showed that 3-PPA had significant inhibitory effects on a series of virulence factors regulated by QS at 12.5, 25.0, and 50.0 μg/mL. Notably, 3-PPA occluded the QS system to reduce virulence and pathogenicity of S. marcescens NJ01 infection as an adjuvant of the antibiotics.

Serratia marcescens NJ01 bacteria can produce thick biofilms, which may allow bacteria to escape from harmful elements and develop drug resistance. In this study, 3-PPA inhibited S. marcescens NJ01 biofilm formation, as confirmed by SEM and CLSM. After 3-PPA exposure, originally tightly connected biofilms became dispersed and structurally disrupted, consistent with the results of QS-related gene mutations in S. marcescens NJ01. Furthermore, S. marcescens NJ01 mutants also exhibit deficiencies in biofilm formation as well as virulence factors (prodigiosin, hemolysin, protease, lipase, and swarming motility) (14, 30). Jayathilake et al. (31) also demonstrated that inhibition of QS can affect bacterial competition and biofilm formation in mixed bacterial strains. Here, 3-PPA treatment resulted in a reduction in biofilm formation with a scattered and flattened appearance that was similar to that formed by QS-deficient mutants and consistent with the QS inhibitory activity of hordenine against S. marcescens NJ01 (15). As 3-PPA inhibited biofilm formation and reduced virulence factor activity in S. marcescens NJ01, we speculated that it may also enhance the effectiveness and sensitivity of S. marcescens NJ01 to antibiotics. Therefore, we further examined the inhibitory effects of 3-PPA (50.0 μg/mL) in combination with the antibiotic ofloxacin (0.2 μg/mL) against preformed biofilms of S. marcescens NJ01. The MIC of ofloxacin against S. marcescens NJ01 is 4 μg/mL, thus we selected a concentration below the MIC (0.2 μg/mL) to test the effects in combination with 3-PPA. The SEM and CLSM results showed that the S. marcescens NJ01 biofilm was tightly connected and thick when ofloxacin (0.2 μg/mL) was used alone and was dense and sheet-like when 3-PPA (50.0 μg/mL) was used alone. However, the combination of 3-PPA and ofloxacin nearly destroyed the biofilm, with only a single colony remaining. These results indicated that 3-PPA had a disruptive effect on formed biofilms when used in combination with ofloxacin. Pan et al. (32) also found that the QSI BF8 can reverse the antibiotic tolerance of P. aeruginosa persister cells. These findings indicate that QSIs could be applied as antibiotic adjuvants to treat drug-resistant bacterial infections. We found that 3-PPA increased the susceptibility of S. marcescens NJ01 to ofloxacin, which has promising implications for anti-biofilm contamination (33). In addition, qRT-PCR identified genes associated with virulence factors and biofilms, consistent with the inhibitory effects of 3-PPA on virulence factor secretion and biofilm formation. Notably, downregulation of *fimA*, *fimC*, *pigP*, *flC*, *flhD*, *bmsB*, and *sodB* by 3-PPA appeared to be the main reason for the decrease in adhesion capacity, biofilm formation, and swarming motility. As shown in Fig. S3 and Table S2 and S3 in Supplemental File 1, purines, and pyrimidines, which are microbial growth factors, were downregulated, indicating that 3-PPA treatment can disrupt the metabolic pathways of purines and pyrimidines and thereby inhibit the growth of microorganisms (34). In addition, several amino acids, such as alanine, aspartate, lysine, arginine, proline, tryptophan, histidine, and glutamate, were vital in QS-related protein synthesis and decreased after 3-PPA treatment. The tricarboxylic acid (TCA) cycle is the most important metabolic pathway providing energy to organisms (35). The amino acid, sphingolipids, glyoxylate, and dicarboxylate pathways are closely related to the tricarboxylic acid (TCA) cycle (36). This indicates that 3-PPA treatment can lead to disruption of the TCA cycle, thereby disrupting microbial energy metabolism and increasing pathogenic dysfunction. In the current study, both qRT-PCR and metabolome analysis showed that inhibition of QS caused an increase in oxidative damage *in vivo* in S. marcescens NJ01, leading to cell membrane disruption, abnormal TCA cycling, and inadequate energy supply. Supply and anaerobic cellular respiration are intensified to increase the energy. The harmful components produced, such as ethanol, further exacerbate the cellular damage, resulting in dysfunctional proteins, increased catabolism, disrupted nucleic acid metabolism, and reduced growth factor content. Ultimately, 3-PPA exposure resulted in dysfunctional physiology and diminished pathogenicity of S. marcescens NJ01.

When present in the form of biofilms, bacteria can tolerate high concentrations of antibiotics and fungicides. Furthermore, uncontrolled use of antimicrobials has led to a rise in multidrug resistance in pathogenic bacteria. Bacteria become incredibly strong in biofilm form, making antibiotic treatment unsuccessful (37). Bacterial pathogens have evolved many antibiotic-resistant systems to survive, resulting in drug resistance and increased death yields. In recent years, researchers have increasingly explored alternative therapeutic strategies to effectively treat pathogen virulence and biofilm formation to overcome the drawbacks of conventional antimicrobial therapies. Unlike antibiotic therapy, resistance to antitoxic therapeutic agents is very low as they specifically attenuate the virulence pathway, rather than the underlying metabolic activity of the pathogen (38). Thus, interfering with the QS system of bacterial pathogens to reduce drug resistance is considered a promising option. We showed that 3-PPA in combination with antibiotics can improve the sensitivity of antibiotics against S. marcescens NJ01. This has significance for reducing the antibiotic dose and use and as a potential anti-biofilm and anti-toxic agent. Overuse of conventional antibiotic treatment has led to the emergence of antibiotic resistance in pathogenic bacteria in the environment.

## MATERIALS AND METHODS

### Strains and reagents.

The S. marcescens NJ01 strain was a kind gift from Yong Yu Li (Fujian Agriculture and Forestry University, China). S. marcescens NJ01, a pathogenic bacterium isolated from diseased tomato leaves, can cause infection in people who consume infected tomatoes. Both 3-PPA and ofloxacin were purchased from Shanghai Yuanye Biotechnology Co., Ltd. (Shanghai, China).

### MIC and growth curve.

The MICs of 3-PPA and quinolone antibiotics (ofloxacin) (Shanghai Yuanye Biotechnology Co., Ltd., Shanghai, China) were determined following the method developed by the Clinical Laboratory Standards Committee (CLSI, 2020). The S. marcescens NJ01 strain was inoculated in 5 mL of Luria-Bertani (LB) broth (Qingdao Hope Biotechnology Co., Ltd., Qingdao, China), then incubated at 180 rpm and 28°C for 17 h (overnight). The overnight culture was diluted to an optical density at 620 nm (OD_620_) of 0.05 (13). Subsequently, different concentrations of drugs were added, and dimethyl sulfoxide (DMSO, Saiguo Biotechnology Co., Ltd., Guangzhou, China) was used as a negative control. The culture (200 μL) was added to a 96-well plate (Corning, USA) and incubated at 28°C and 180 rpm for 24 h. Absorbance was determined at 620 nm. All experiments were repeated at least three times.

3-PPA (12.5, 25.0, and 50.0 μg/mL) was added to the diluted solution and cultured at 28°C and 180 rpm for 24 h. DMSO was used as a negative control and did not exceed 1% of the bacterial solution. The culture medium was taken every 3 h to measure OD_620_, and a growth curve was constructed.

### Virulence factor assay.

**(i) Protease activity determination.** Overnight S. marcescens NJ01 seed solution was added to LB broth (1:100, vol/vol) supplemented with 3-PPA (12.5, 25.0, and 50.0 μg/mL), and cultured at 28°C and 180 rpm for 24 h. DMSO served as a negative control. The overnight culture was centrifuged at 4°C and 8 000 rpm for 5 min, with 75 μL of the resulting supernatant then mixed with 125 μL of 2% azocasein solution (dissolved in 25 M Tris-HCl, Sangon Biotech, China). The mixture was incubated at 37°C for 15 min and added to 600 μL of 10% trichloroacetic acid (Shanghai Macklin Biochemical Technology Co., Ltd., China). The reaction was terminated with 1 M NaOH (Saiguo Biotechnology Co., Ltd., Guangzhou, China), and the protease activity was determined at OD_440_.

**(ii) Lipolytic activity determination.** Lipolytic activity was determined using *p*-nitrophenyl palmitate (*p*NPP, Shanghai Macklin Biochemical Technology Co., Ltd., China), as described previously (20). First, 3-PPA (12.5, 25.0, and 50.0 μg/mL) was added to the prepared S. marcescens NJ01 solution and cultivated at 28°C and 180 rpm for 24 h. After culturing, 100 μL of the supernatant was taken and mixed with 900 μL of the buffered substrate (0.3% *p*NPP in isopropanol, 0.2% sodium deoxycholate [Shanghai Macklin Biochemical Technology Co., Ltd., China], and 0.1% gummi arabicum [Aladdin, Shanghai, China] in 50 mM Na_2_PO_4_ buffer [Aladdin, Shanghai, China]). The solution was induced at room temperature for 1 h in the dark with 1 mL of Na_2_CO_3_ (1 M, Aladdin, Shanghai, China), and then added to terminate the reaction. In total, 200 μL of the supernatant obtained by centrifugation (4°C, 12,000 rpm, 10 min) was taken and absorbance was measured at an optical density of 410 nm (OD_410_).

**(iii) Hemolysin assay.** After the bacterial solution was cultured with 3-PPA (12.5, 25.0, and 50.0 μg/mL), the supernatant was obtained by centrifugation (4°C, 3,000 rpm, 10 min). Mouse blood (M5905, Sigma-Aldrich, USA) and heparin sodium (Shanghai Yuanye Biotechnology Co., Ltd., China) were mixed with the supernatant (9/1, vol/vol), followed by incubation at 37°C for 1 h. The supernatant was again centrifuged (4°C, 3,000 rpm, 10 min) and absorbance was determined at OD_530_.

**(iv) Determination of prodigiosin content.** The cultured bacterial solution was taken (1 mL) and centrifuged (4°C, 12,000 rpm, 5 min) to collect bacterial cells. The cells were washed thoroughly with ultrapure water, resuspended, and centrifuged (4°C, 12,000 rpm, 5 min). The cells were then mixed with 1 mL of acidified ethanol (4%; 1 M HCl [Aladdin, Shanghai, China]) to extract pigments. The pigments were determined at OD_534_.

**(v) Swimming assay.** Here, 3-PPA was added to 100 mL of swimming medium (peptone 1%, NaCl 0.5%, and agar 0.3% [Qingdao Hope Biotechnology Co., Ltd., Qingdao, China], pH = 7.0). The final concentrations of 3-PPA were 12.5, 25.0, and 50.0 μg/mL. After mixing, the medium was evenly poured into a Petri dish. We collected 5 μL of the prepared cultured bacterial solution and placed it in the center of the solidified medium. DMSO was used as a negative control. Subsequently, the Petri dish was incubated at 28°C for 24 h to observe the swimming diameter (39).

### Biofilm inhibition.

We added 1 mL of prepared culture medium to a 24-well plate (Corning, USA), followed by 3-PPA (12.5, 25.0, and 50.0 μg/mL). The plate was then cultured at 28°C for 24 h, after which the overnight culture suspension was removed, washed three times with PBS (Saiguo Biotechnology Co., Ltd., Guangzhou, China), and oven-dried at 60°C. Subsequently, MeOH (Aladdin, Shanghai, China) was added to the 24-well plate for 15 min to fix the biofilm. After the methanol completely evaporated to dryness, 500 μL of crystal violet (0.05%, Shanghai Macklin Biochemical Technology Co., Ltd., China) was added to each well for staining, then removed after 15 min. The stained biofilm was washed three times with PBS and dried. EtOH (95%; 1 mL) was used for decolorization. In total, 150 μL of the decolorizing solution was taken and absorbance was determined at OD_570_ (15).

### Biofilm erasure of 3-PPA combined with ofloxacin.

The prepared culture medium was placed in a 24-well plate and cultured at 28°C for 24 h. The overnight culture was then removed, and the biofilm was washed three times with PBS. Both 3-PPA (50.0 μg/mL) and ofloxacin (0.2 μg/mL) were added to fresh LB broth and mixed well. The mixed medium was taken (1 mL) and added to a 24-well plate with culture at 28°C for 24 h. After incubation, the biofilm was processed and quantified according to the Biofilm Inhibition section (40).

### SEM analysis.

**(i) Biofilm inhibition.** A round glass coverslip was placed in a 24-well plate followed by the addition of 1 mL of the prepared culture medium, as well as 3-PPA (12.5, 25.0, and 50.0 μg/mL). The plate was incubated at 28°C for 24 h. Subsequently, the bottom circular coverslip was gently removed with tweezers. PBS was used to remove planktonic bacteria from the coverslips (15).

**(ii) Biofilm erasure.** The prepared culture medium (1 mL) was added to a 24-well plate with a round glass coverslip at the bottom and incubated at 28°C for 24 h. The supernatant medium was removed and washed gently three times with PBS. In addition, 1 mL of fresh LB broth containing 3-PPA (50.0 μg/mL) and ofloxacin (0.2 μg/mL) was added to the 24-well plate, followed by incubation at 28°C for 24 h. The circular coverslip was gently retrieved with tweezers and washed to remove planktonic bacteria using PBS.

The treated coverslip, which contained a biofilm, was then fixed with 2.5% glutaraldehyde for 10 h, dehydrated with EtOH (50%, 60%, 70%, 80%, 90%, and 100%), and freeze-dried for 2 h when EtOH was slightly dry. Biofilm morphology was observed by SEM (JSM6360, JEOL, Tokyo, Japan) after gold spraying (41).

### CLSM analysis.

The S. marcescens NJ01 strain was cultured as described above. Planktonic bacteria on the circular coverslips were washed with PBS after incubation. Subsequently, acridine orange (5 μL, Shanghai Macklin Biochemical Technology Co., Ltd., China) and ethidium bromide (5 μL, Aladdin, Shanghai, China) was added dropwise to the glass slide to stain the biofilm for 5 min. Excess dye was washed using PBS, and the coverslips were observed using a confocal laser scanning microscopy (CLSM, Zeiss LSM 700, Carl Zeiss, Jena, Germany) (42).

### Quantitative real-time PCR.

The cultured S. marcescens NJ01 bacterial solution was centrifuged at 4°C and 12,000 rpm for 15 min to collect bacterial cells, then washed three times with precooled PBS. Total RNA was extracted using a Tsingke RNA Extraction kit (Tsingke Biological Technology Co., Ltd., Beijing, China). The primers used in qRT-PCR are shown in Table 1, with *rpsl* used as the internal reference gene. Primers were synthesized by Tsingke Biological Technology Co., Ltd., and genomic DNA was removed and amplified by reverse transcription using a Goldenstar RT6 cDNA Synthesis kit (Tsingke Biological Technology Co., Ltd., Beijing, China). Absolute quantitative detection was performed using the SYBR green I (Aladdin, Shanghai, China) dye method.

**TABLE 1:** PCR primers for qRT-PCR

Genes	Primer direction	Sequence (5′–3′)	Amplicon size (bp)
*fimA*	Forward	TTAGCCTGGAGAAATGTGAAGC	145
	Reverse	GGCAGAGTAGAGCCGTTGTTAT	
*fimC*	Forward	AGCAGTTCAACACCTCCTTCAT	216
	Reverse	CGGATATTTACCCGGCAGA	
*bsmB*	Forward	CGGAAGTGACGCTGGAACACG	150
	Reverse	TGCTGCTGTTGATGGTGTAATCGG	
*pigP*	Forward	CGTCCGCCGGTACTGATT	132
	Reverse	GTAACCCAGGTATTGCACGGT	
*flhC*	Forward	CATTTATCACCCACGCGCAC	102
	Reverse	CCAGTTGCGGCGAAAGTTTA	
*sodB*	Forward	CTGCTGACCGTTGACGTGTGG	108
	Reverse	CGCTGCGAAGGTCCAGTTGAC	
*flhD*	Forward	GTCCGCAATGTTTCGCCTG	123
	Reverse	TCGTTAAAGCGGAAGTGGCA	
*rplU*	Forward	CAACACCGAGTAAGCGAAGG	131

### Intracellular metabolite analysis.

The bacterial cells were prepared as described above. The cultured S. marcescens NJ01 bacterial solution was centrifuged at 4°C and 10,000 rpm for 10 min to obtain bacterial cells, then washed three times with PBS. The cells were homogenized to obtain metabolites. The metabolites were dissolved in methanol and analyzed by liquid chromatography-tandem mass spectrometry (LC-MS/MS) using an ultrahigh-performance liquid chromatography (UHPLC) system (Vanquish, Thermo Fisher Scientific) with a ultra-performance liquid chromatography and ethyl bridge hybridization (UPLC BEH) Amide column (2.1 mm × 100 mm, 1.7 μm) coupled to a Q-Exactive HFX mass spectrometer (Orbitrap MS, Thermo). The mobile phases were 25 mmol/L ammonium acetate and 25 mmol/L ammonia hydroxide in water (pH = 9.75) (A) and acetonitrile (B). The auto-sampler temperature was set to 4°C and the injection volume was set to 3 μL. The Q-E HFX mass spectrometer was used to acquire MS/MS spectra in the information-dependent acquisition (IDA) mode under the control of the acquisition software (Xcalibur, Thermo), which continuously evaluates the full-scan MS spectrum. The electrospray ionization (ESI) source conditions were set as follows: sheath gas flow rate of 30 Arb, Aux gas flow rate of 25 Arb, capillary temperature of 350°C, full MS resolution of 60 000, MS/MS resolution of 7,500, collision energy of 10/30/60 in NCE mode, and spray voltage of 3.6 kV (positive) or −3.2 kV (negative), respectively.

Metabolites were assigned based on publicly accessible metabolomic databases (Human Metabolome Database [HMDB], http://www.hmdb.ca/; MassBank, http://www.massbank.jp/) and the BiotreeDB in-house MS2 database (Biotree Biochemical Technology Co., Ltd., Shanghai, China) (43). To further interpret the biological significance of metabolites, metabolic pathway analyses were performed using the online analysis platform in MetaboAnalyst v5.0 (https://metaboanalyst.ca/). Kyoto Encyclopedia of Genes and Genomes (KEGG; http://www.kegg.jp/kegg/pathway.html) enrichment analysis was also conducted using MetaboAnalyst.

### Statistical analysis.

All assays were performed in triplicate. Results were presented as mean ± standard deviation (SD). Two-way analysis of variance (ANOVA) was applied to assess significance using GraphPad Prism v8. Differences were considered statistically significant at *, *P* < 0.05; **, *P* < 0.01; and ***, *P* < 0.001.
