# Supplementary material for: 3-Phenylpropan-1-Amine Enhanced Susceptibility of Serratia marcescens to Ofloxacin by Occluding Quorum Sensing
Source: Microbiol Spectr. 2022 Aug 16;10(5):e01829-22. doi: 10.1128/spectrum.01829-22 (PMC9603881; doi:10.1128/spectrum.01829-22)
Supplement: Supplemental file 1 — Fig. S1 to S3 and Tables S1 to S3. Download spectrum.01829-22-s0001.pdf, PDF file, 0.5 MB [file spectrum.01829-22-s0001.pdf]

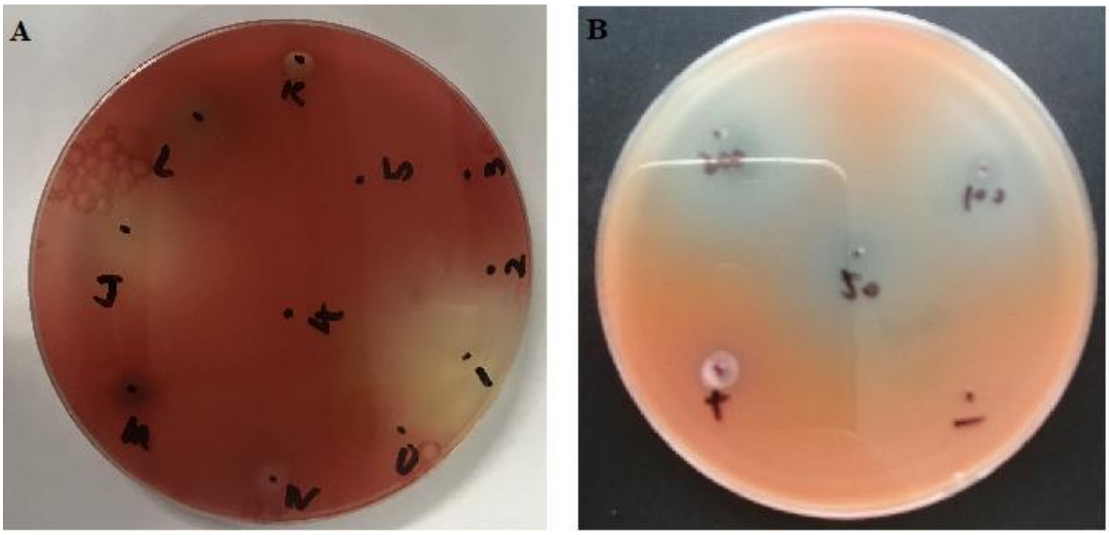

Fig. S1 Screening of quorum sensing inhibitor for *S. marcescens* NJ01.

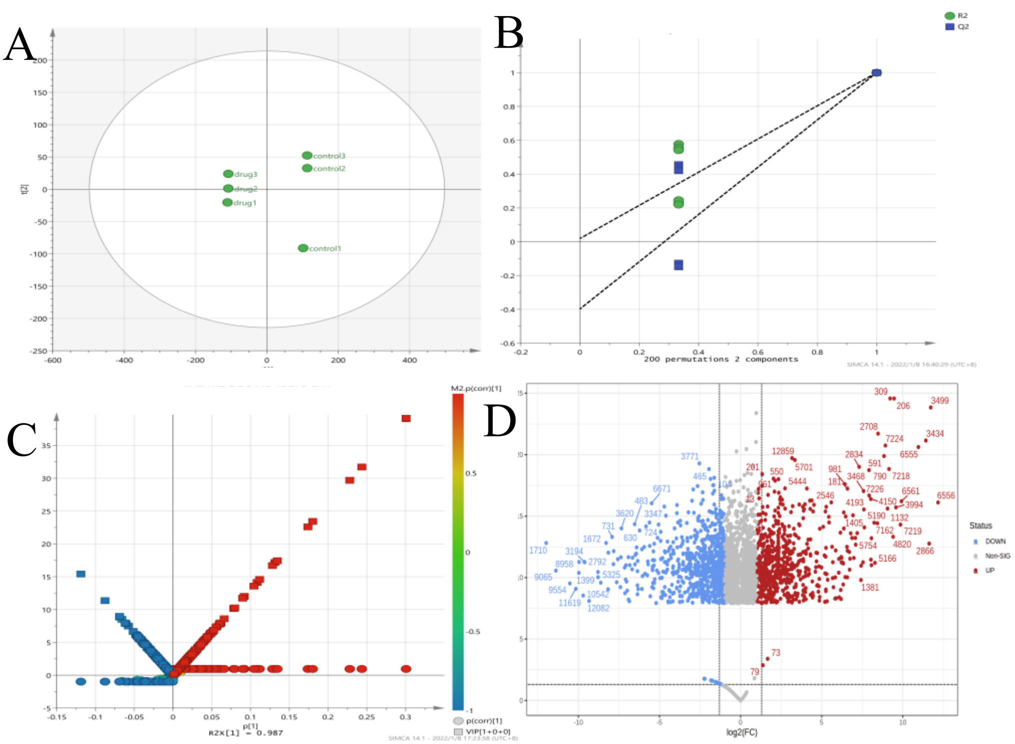

Fig. S2 Screening of differential metabolites (A) Score scatter plot of PCA model of group 3-PPA vs control; (B) Permutation test of OPLS-DA model for group 3-PPA vs control; (C) VIP and S-plot of OPLS-DA for group 3-PPA vs control; (D) volcano plot for group 3-PPA vs control.

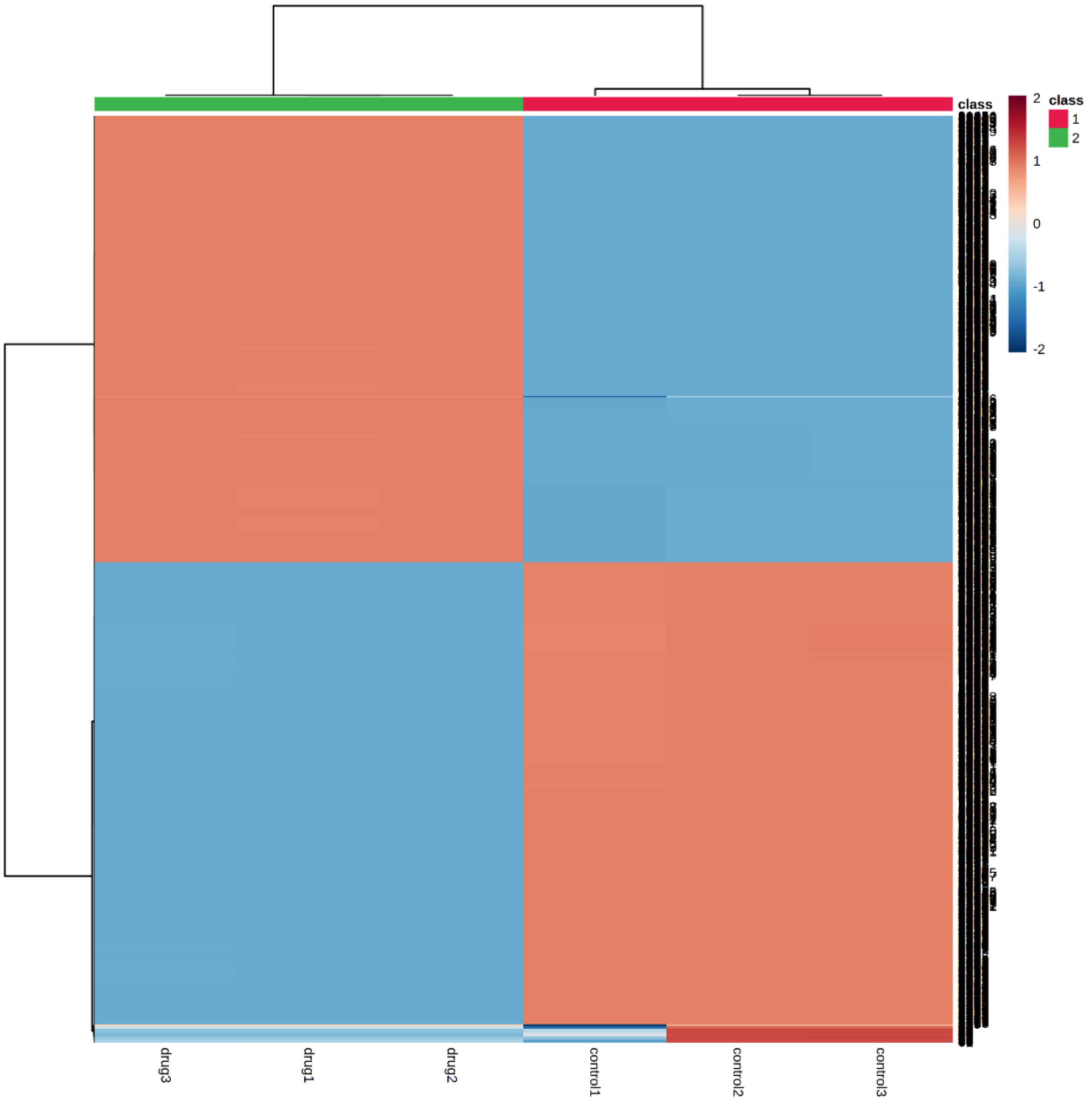

Fig. S3 Heatmap of hierarchical clustering analysis for group 3-PPA treatment vs control.

Table S1 Effects of hordenine analogues on quorum sensing in *S. marcescens* NJ01

Drug	QS inhibition
3-phenylpropan-1-amine (3-PPA)	+
4-(2-aminoethyl)-phenol	-
4-(2-(methylamino)-ethyl)-phenol	-
N-methyl-2-phenylethanamine	-
2-phenylethanamine	-
(*E*)-*N*-methyl-3-phenylprop-2-en-1-amine	-
N-methyl-3-phenylpropanamide	-

L-phenylalanine	-
3-phenylpropanamide	-
cinnamamide	-
N-methyl-3-phenylpropan-1-amine	+

$^{+}$ represents QS inhibitory activity, $^{-}$ is inactive

Table S2 Identified and changes involved in intracellular metabolites

No.	Metavolites	FC[a]	Log^2 (FC)[b]	*P*
1	2-Pyrrolidinone	2.9303	1.551	6.83E-16
2	Acetylhydrazine	0.3075	-1.7013	3.04E-18
3	1-deoxy-1-(N6-lysino)-D-fructose	2.1085	1.0762	1.27E-17
4	Dimethylglycine	0.28008	-1.8361	4.51E-16
5	Sarcosine	0.27694	-1.8524	9.34E-19
6	Aminoadipic acid	0.32447	-1.6239	7.55E-19
7	Sphinganine	0.30482	-1.714	1.45E-17
8	2-Ethoxy-5-methylpyrazine	0.45301	-1.1424	1.06E-15
9	Kynurenic acid	0.12554	-2.9937	5.52E-17
10	Glycyl-Valine	0.136	-2.8783	5.31E-16
11	Propylpyrazine	2.4329	1.2827	2.35E-15
12	5-Ethyl-2-(1-pyrrolidinyl)-2-cyclopenten-1-one	0.18227	-2.4559	1.56E-14
13	N-Acetylcadaverine	0.48119	-1.0553	3.82E-17
14	Methamphetamine	96.227	6.5884	5.78E-18
15	7-Aminonitrazepam	2.1735	1.12	9.13E-15
16	L-Arginine	2.5136	1.3297	3.86E-19
17	3-Ethylidenehexahydropyrrolo[1,2-a]pyrazine-1,4-dione	0.41573	-1.2663	3.52E-16
18	Amphetamine	705.7	9.4629	2.69E-25
19	N-Ethylglycine	0.43449	-1.2026	3.48E-15
20	(23S,24S)-17,23-Epoxy-24,29-dihydroxy-27-norlanost-8-ene-3,15-dione	12.096	3.5964	4.56E-15
21	3-Methylhistidine	2.5949	1.3757	5.35E-16
22	Prostaglandin I2	0.4326	-1.2089	1.31E-15
23	Pyridoxal	2.6095	1.3838	8.96E-15
24	5-Methyl-2-propyloxazole	0.35363	-1.4997	7.19E-16
25	Acrimarine N	23.002	4.5237	1.20E-16
26	Dodecyl gallate	3.5401	1.8238	1.58E-14
27	4-(Hydroxymethyl) benzenediazonium	595.41	9.2177	2.65E-25
28	N-Nitroso-pyrrolidine	0.24758	-2.014	2.07E-15
29	Xanthurenic acid	0.29822	-1.7456	3.70E-15
30	Methionine sulfoxide	0.41202	-1.2792	5.92E-16
31	Isoleucyl-Alanine	0.44521	-1.1674	4.69E-16
32	Seryltyrosine	0.34348	-1.5417	4.51E-15
33	Octadecylamine	0.39055	-1.3564	2.17E-15
34	Polyoxyethylene dioleate	0.05944	-4.0724	9.89E-15
35	gamma-Aminobutyric acid	0.21581	-2.2121	5.04E-15
36	Arginyl-Methionine	5.0007	2.3221	5.43E-16
37	Aspartyl-Leucine	0.25858	-1.9513	1.47E-19

38	Gibberellin A70	0.010804	-6.5323	4.50E-15
39	Hydroxyprolyl-Leucine	0.41678	-1.2626	1.12E-14
40	Vinylacetylglycine	0.34844	-1.521	2.55E-16
41	Isopentyl beta-D-glucoside	5.0439	2.3345	9.84E-19
42	N-Methylcalystegine B2	14.254	3.8333	1.72E-16
43	Pipereicosalidine	5.001	2.3222	2.65E-15
44	1-(2,3-Dihydro-5,6-dimethyl-1H-pyrrolizin-7-yl) ethanone	459.05	8.8425	1.30E-20
45	1-(Methylsulfanyl)-1-oxopropan-2-yl acetate	6.0907	2.6066	6.48E-15
46	Cohibin A	0.27244	-1.876	6.70E-15
47	N, N-Diethylbenzeneacetamide	89.251	6.4798	1.07E-15
48	Brevianamide B	82.145	6.3601	4.99E-16
49	2-Methoxy-3-(1-methylpropyl) pyrazine	4.4824	2.1643	2.32E-15
50	Dibutyl malate	0.013345	-6.2275	1.47E-14
51	Cohibin C	5.4702	2.4516	2.94E-17
52	8-Hydroxycarteolol	2.1899	1.1309	1.58E-14
53	Nor-psi-tropine	4.5901	2.1985	3.93E-15
54	PE (15:0/15:0)	0.31522	-1.6656	4.90E-15
55	PE (20:1(11Z)/15:0)	0.41603	-1.2652	5.93E-15
56	PE (20:1(11Z)/14:0)	0.017563	-5.8314	6.78E-15
57	PC(P-18:1(11Z)/16:0)	0.33521	-1.5769	5.03E-16
58	3'-Deoxydihydrostreptomycin 6,3"-bis-phosphate	0.3481	-1.5224	6.34E-14
59	4,11-Dichloro-5,12-dihydroquino[2,3-b] acridine-7,14-dione	2.5658	1.3594	1.80E-16
60	[2,6-dihydroxy-3-(3-phenylpropanoyl) phenyl] oxidanesulfonic acid	0.24655	-2.0201	3.89E-14
61	1-Methyladenosine	0.48677	-1.0387	6.57E-16
62	3-(ADP)-glycerate	29.492	4.8823	1.05E-14
63	Caffeoyl aspartic acid	0.041094	-4.6049	9.12E-16
64	Pyrifenox	2.8643	1.5182	2.04E-15
65	Clomeprop	12.238	3.6133	1.13E-16
66	2-amino-4-({1-[(carboxymethyl)-C-hydroxycarbonimidoyl]-2-({2,4-dihydroxy-5-[(2E)-3-(4-hydroxyphenyl) prop-2-enoyl]-3,6-dioxocyclohexa-1,4-dien-1-yl}sulfanyl) ethyl}-C-hydroxycarbonimidoyl)butanoic acid	0.10049	-3.3149	3.61E-15
67	Chloramphenicol 3-acetate	5.0011	2.3222	3.82E-15
68	Iridin	0.038429	-4.7017	1.67E-16
69	CMP	2.3715	1.2458	5.43E-14
70	Kolaflavanone	4.9384	2.304	3.61E-14
71	Debromohymenialdisine	0.44037	-1.1832	7.92E-16

72	Isopentenyl phosphate	0.36896	-1.4385	8.42E-12
73	Puerarin xyloside	3.699	1.8871	1.85E-14
74	Pradimicinone I	4.7563	2.2498	3.38E-13
75	Succinic anhydride	0.16831	-2.5708	1.36E-12
76	2-Maleylacetate	0.1714	-2.5446	5.09E-20
77	Iridin	8.6228	3.1082	4.72E-16
78	5''-Phosphoribostamycin	7.9657	2.9938	1.00E-13
79	Nitrofen	0.29169	-1.7775	8.37E-13
80	2-({2-[4-(1,2-dihydroxyethyl)-5,11,12,13-tetrahydroxy-8-oxo-3,7-dioxatricyclo[7.4.0.0trideca-1(13),9,11-trien-10-yl]-3,4,8,9,10-pentahydroxy-6-oxo-6H-benzo[c]chromen-1-yl} formamido) acetic acid	0.43467	-1.202	1.37E-12
81	Dihydrostreptomycin 3'alpha,6-bisphosphate	0.40676	-1.2977	2.91E-12
82	CMP	5.9041	2.5617	4.65E-15
83	ADP	17.995	4.1695	4.14E-13
84	Oxolinic acid	3.1264	1.6445	4.71E-13
85	Se-Propenylselenocysteine Se-oxide	3.4801	1.7991	3.22E-14
86	2-({2-[4-(1,2-dihydroxyethyl)-5,11,12,13-tetrahydroxy-8-oxo-3,7-dioxatricyclo[7.4.0.0trideca-1(13),9,11-trien-10-yl]-3,4,8,9,10-pentahydroxy-6-oxo-6H-benzo[c]chromen-1-yl} formamido) acetic acid	0.33717	-1.5685	4.20E-14
87	Calcium propiote	675.96	9.4008	4.79E-14
88	1,1-Dichloro-2,2-diphenylethane	4.8759	2.2857	1.01E-12
89	Trinitrotoluene	0.13945	-2.8422	1.38E-13
90	3-Phosphoglycerol-glutathione	7.1661	2.8412	7.79E-13
91	Maysin 3'-methyl ether	0.043961	-4.5076	1.18E-13
92	2-amino-4-({1-[(carboxymethyl)-C-hydroxycarbonimidoyl]-2-({2,4-dihydroxy-5-[(2E)-3-(4-hydroxyphenyl) prop-2-enoyl]-3,6-dioxocyclohexa-1,4-dien-1-yl}sulfanyl) ethyl}-C-hydroxycarbonimidoyl)butanoic acid	0.04352	-4.5222	8.45E-12
93	2,5-Furandicarboxylate	0.29774	-1.7479	3.06E-12
94	Proanthocyanidin A2	0.37264	-1.4241	1.31E-13
95	CMP	8.4089	3.0719	3.78E-14
96	2-({2-[4-(1,2-dihydroxyethyl)-5,11,12,13-tetrahydroxy-8-oxo-3,7-dioxatricyclo[7.4.0.0trideca-1(13),9,11-trien-10-yl]-3,4,8,9,10-pentahydroxy-6-	9.7154	3.2803	1.06E-14

	oxo-6H-benzo[c]chromen-1-yl}formamido) acetic acid			
97	Kolaflavanone	0.12126	-3.0439	1.07E-12
98	Iridin	0.077792	-3.6842	2.83E-13
99	Apigenin 7-O-[beta-D-apiosyl-(1->2)-beta-D-glucoside]	0.19778	-2.3381	4.87E-12
100	Maysin 3'-methyl ether	0.029994	-5.0592	4.83E-15
101	2-amino-4-({1-[(carboxymethyl)-C-hydroxycarbonimidoyl]-2-({2,4-dihydroxy-5-[(2E)-3-(4-hydroxyphenyl) prop-2-enoyl]-3,6-dioxocyclohexa-1,4-dien-1-yl} sulfanyl) ethyl}-C-hydroxycarbonimidoyl) butanoic acid	0.02496	-5.3242	1.76E-13
102	Amaroswerin	0.056244	-4.1522	1.31E-13
103	Trimethylselenonium	2.6529	1.4075	2.86E-13
104	Chloropropylate	3.6111	1.8525	5.71E-13
105	Tetracozole	7.8972	2.9813	6.62E-15
106	Amaroswerin	0.025042	-5.3195	3.86E-12
107	2-amino-4-({1-[(carboxymethyl)-C-hydroxycarbonimidoyl]-2-({2,4-dihydroxy-5-[(2E)-3-(4-hydroxyphenyl) prop-2-enoyl]-3,6-dioxocyclohexa-1,4-dien-1-yl} sulfanyl) ethyl}-C-hydroxycarbonimidoyl)butanoic acid	0.035499	-4.8161	2.58E-12
108	Melizame	0.43433	-1.2031	5.17E-13
109	Dihydrostreptomycin 3'alpha,6-bisphosphate	0.47374	-1.0778	9.93E-14
110	Threote	4.3653	2.1261	1.30E-18
111	2-({2-[4-(1,2-dihydroxyethyl)-5,11,12,13-tetrahydroxy-8-oxo-3,7-dioxatricyclo[7.4.0.0trideca-1(13),9,11-trien-10-yl]-3,4,8,9,10-pentahydroxy-6-oxo-6H-benzo[c]chromen-1-yl} formamido) acetic acid	0.014755	-6.0827	4.94E-13
112	Dihydrostreptomycin 3'alpha,6-bisphosphate	0.011762	-6.4097	3.47E-13
113	Melizame	0.26565	-1.9124	9.74E-13
114	Chlorfenson	6.6832	2.7405	2.42E-16
115	3,8-Diglucosyldiosmetin	0.21748	-2.201	4.47E-13
116	Gallocatechin-(4alpha->8)-epigallocatechin	0.1981	-2.3357	2.15E-12
117	6-{4-[(1E)-3-[(6-{[3,4-dihydroxy-2,5-bis(hydroxymethyl)oxolan-2-yl] oxy}-3,4,5-trihydroxyoxan-2-yl) methoxy]-3-	0.17907	-2.4814	6.37E-13

	oxoprop-1-en-1-yl]-2-hydroxyphenoxy}-3,4,5-trihydroxyoxane-2-carboxylic acid			
118	Hypoxanthine	242.73	7.9232	2.09E-17
119	4-(3-methylbut-2-en-1-yl)-8,17-dioxatetracyclo [8.7.0.0,.0,] heptadeca-1(10),2(7),3,5,11(16),12,14-heptaene-3,5,14-triol	0.24325	-2.0395	7.51E-12
120	Angustine	0.18734	-2.4162	2.92E-12
121	2-({2-[4-(1,2-dihydroxyethyl)-5,11,12,13-tetrahydroxy-8-oxo-3,7-dioxatricyclo[7.4.0.0trideca-1(13),9,11-trien-10-yl]-3,4,8,9,10-pentahydroxy-6-oxo-6H-benzo[c]chromen-1-yl} formamido) acetic acid	0.045936	-4.4442	3.88E-12
122	3-(Uracil-1-yl)-L-alanine	0.47989	-1.0592	2.99E-14
123	Monomethyl phenylphosphonate	0.19032	-2.3935	9.96E-14
124	Succinic anhydride	0.48771	-1.0359	6.96E-12
125	4-Bromo-3,5-cyclohexadiene-1,2-dione	0.38098	-1.3922	3.06E-14
126	2-Oxoadipate	0.21349	-2.2278	2.89E-12
127	2-Hydroxymucote semialdehyde	0.34831	-1.5216	1.85E-12
128	2-Oxoadipate	0.36786	-1.4428	7.39E-16
129	Monodehydroascorbate	3.4591	1.7904	4.54E-14
130	4-Methylene-L-glutamate	0.20089	-2.3155	2.51E-12

[a]Multiple changes of intracellular metabolites of *Serratia marcescens* NJ01 after 3-phenylpropan-1-amine action.

Red (+) and blue (-) represent the increased and decreased metabolites, respectively, in 3-phenylpropan-1-amine-treated group.

Table S3 Key metabolic pathways for comparison of 3-PPA treatment

Pathway name	Total	Hits	Raw *p*	Holm adjust	FDR	Impact
Alanine, aspartate and glutamate metabolism	22	3	0.35736	1	1	0.45324
Butanoate metabolism	14	2	0.39736	1	1	0.4
Sphingolipid metabolism	13	2	0.36126	1	1	0.35715
beta-Alanine metabolism	11	2	0.28709	1	1	0.33333
Arginine biosynthesis	18	3	0.24699	1	1	0.29
Lysine biosynthesis	16	3	0.19408	1	1	0.27906
Vitamin B6 metabolism	11	1	0.67418	1	1	0.20513
Purine metabolism	62	5	0.73466	1	1	0.19205
Cysteine and methionine metabolism	41	3	0.77616	1	1	0.13706
Pantothenate and CoA biosynthesis	20	2	0.59	1	1	0.12467
Glycerophospholipid metabolism	32	1	0.96328	1	1	0.11329
Arginine and proline metabolism	25	5	0.08372	1	1	0.1118

Pyrimidine metabolism	34	1	0.97026	1	1	0.01998
Glycosylphosphatidylinositol (GPI)-anchor biosynthesis	14	1	0.76065	1	1	0.00399
